# Using Jupiter’s gravitational field to probe the Jovian convective dynamo

**DOI:** 10.1038/srep23497

**Published:** 2016-03-23

**Authors:** Dali Kong, Keke Zhang, Gerald Schubert

**Affiliations:** 1 Key Laboratory of Planetary Sciences, Shanghai Astronomical Observatory, Chinese Academy of Sciences, Shanghai 200030, China; 2 Center for Geophysical and Astrophysical Fluid Dynamics, University of Exeter, EX4 4QE, UK; 3 Lunar and Planetary Science Laboratory, Macau University of Science and Technology, Macau, China; 4 Department of Earth, Planetary and Space Sciences, University of California, Los Angeles, CA 90095-1567, USA

## Abstract

Convective motion in the deep metallic hydrogen region of Jupiter is believed to generate its magnetic field, the strongest in the solar system. The amplitude, structure and depth of the convective motion are unknown. A promising way of probing the Jovian convective dynamo is to measure its effect on the external gravitational field, a task to be soon undertaken by the Juno spacecraft. We calculate the gravitational signature of non-axisymmetric convective motion in the Jovian metallic hydrogen region and show that with sufficiently accurate measurements it can reveal the nature of the deep convection.

Jupiter possesses the strongest planetary magnetic field in the solar system, more than ten times larger than that of Earth^1^. It is widely accepted that the Jovian magnetic field is generated by convection-driven motion in the deep metallic hydrogen region of the planet^2^,^3^. However, we know very little about the amplitude and structure of the convective motion, and we do not even know the depth at which the Jovian dynamo operates^3^. Magnetohydrodynamic dynamo processes taking place in the Jovian deep interior are highly complex: thermal buoyancy forces in the metallic region drive convective motion which is strongly controlled by Coriolis forces and which, through magnetic induction, converts the mechanical energy of the fluid motion into the ohmic dissipation of the magnetic field. Though progress has been made in modeling the Jovian convective dynamo^4^,^5^, achieving the realistic physical parameters probably never will be possible and extrapolating the convective dynamo from a numerically accessible model over many orders of magnitude would lead to a high uncertainty. Here we propose that high-precision measurements of the Jovian gravitational field can provide a window into the phenomenon.

Using a fully three-dimensional finite element model we calculate the external gravitational signature of non-axisymmetric motions in the dynamo region of Jupiter’s deep interior. Juno, or some future Jupiter orbiter, could measure this signature thereby sounding the deep interior. The model is characterized by the depth *H* to the top of the dynamo region, the typical horizontal length scale 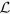 of the convection and its amplitude 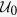. It assumes that Jupiter is isolated, rotates rapidly about the symmetry axis, and consists of a compressible barotropic fluid (a polytrope of index unity) whose density is a function only of pressure^6^,^7^. The axisymmetric zonal winds at Jupiter’s cloud level are confined in the outer molecular layer with equatorial thickness *H* and the underlying metallic hydrogen region has equatorial radius (*R*_*e*_ − *H*), where *R*_*e*_ is Jupiter’s equatorial radius. The Jovian magnetic field is generated by a parameterized convection with amplitude 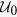 and horizontal length scale 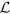 in the metallic hydrogen region. The horizontal scale 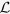 is related to an azimuthal wavenumber *m*_0_. Although the Jovian magnetic field does not explicitly enter the gravitational sounding model, 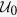 and *m*_0_ reflect the properties of the Jovian convective dynamo^8^,^9^. For example, a small azimuthal wavenumber *m*_0_ = O(1) (large horizontal scale) dominating the structure of the fluid motion could be indicative of a strong field Jovian dynamo with a large invisible toroidal component of the internal field. A large value of *m*_0_ (small horizontal scale) could be indicative of a weak field Jovian dynamo. Additional model details can be found in the Methods section.

## Results

Prior to solving for the external gravitational signature of the nonaxisymmetric motion in the metallic dynamo region we must first determine the nonspherical shapes of both the outer bounding surface and the interface between the metallic region and the molecular envelope, along with the internal density distribution and its gravitational field. Because of axisymmetry, the zonal-wind-induced density anomalies only modify the zonal gravitational coefficients^6^,^10^. The convection-induced gravitational perturbation is non-axisymmetric, and so it can be readily discerned from the gravitational anomalies caused by the fast zonal winds.

We know very little about the typical amplitude 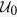 and the spatial structure of the convective motion taking place in the deep interior of Jupiter^3^. It is believed that 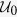 would be much smaller than the speed of the fast zonal winds which is of O(100) m/s. Estimates of 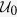 in the metallic hydrogen-helium region vary from 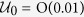 m/s to 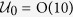 m/s^4^,^5^. This uncertainty is because we cannot access the Jovian physical parameters via direct numerical simulation and an extrapolation on the basis of scaling laws over many orders of magnitude is highly unreliable^3^,^11^. In our current solutions of gravitational sounding, we take the amplitude 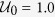 m/s because the governing equations are linear and, hence, the result for other values of the amplitude can be obtained by rescaling.

**Figure 1:**
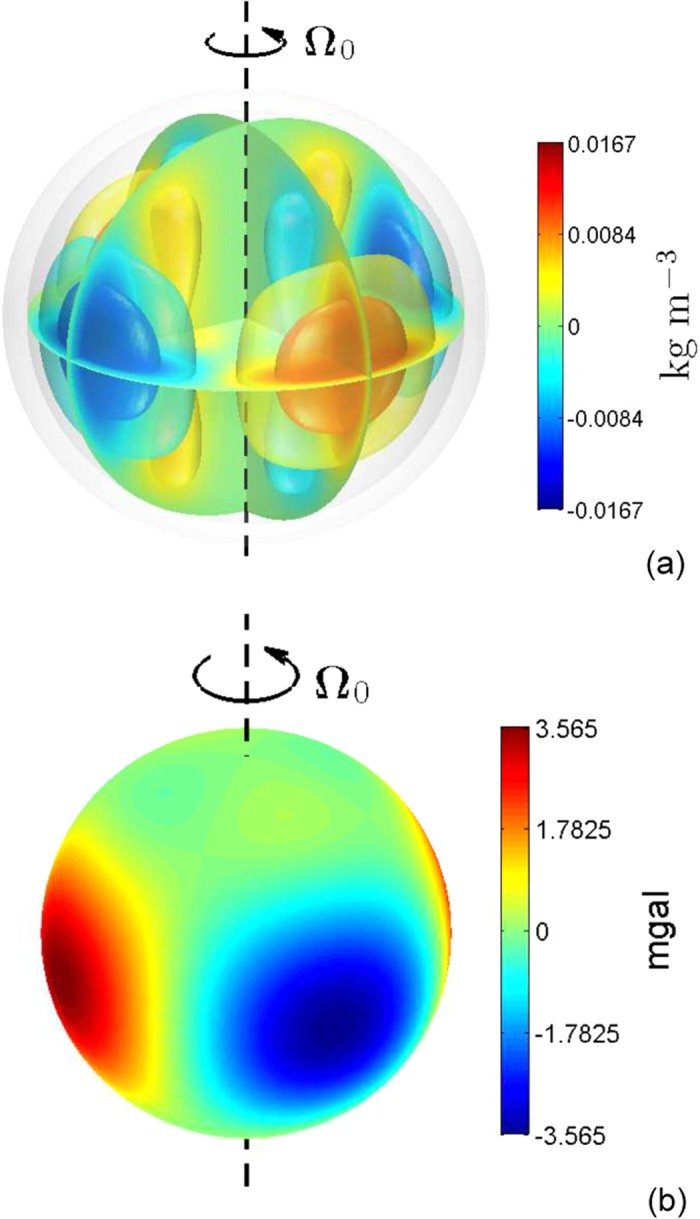
(**a**) Density anomalies in the metallic hydrogen region induced by convective motion with amplitude 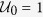 m/s and azimuthal wavenumber *m*_0_ = 2 in the rotationally distorted Jupiter and (**b**) the corresponding radial gravitational anomalies near the outer surface of Jupiter. The metallic-molecular interface is located at equatorial radius 0.9*R*_*e*_.

We first consider a solution with a large horizontal scale of the convective flow marked by the azimuthal wavenumber *m*_0_ = 2, which may correspond to a strong field Jovian dynamo. A three-dimensional view of the density anomalies induced by the convective motion in the non-spherical metallic dynamo region with depth parameter *H* = 0.1*R*_*e*_ is shown in Fig. 1(a) and the corresponding radial gravitational anomalies near the outer bounding surface of Jupiter are shown in in Fig. 1(b). It can be seen, as expected, that the density anomalies are also characterized by azimuthal wavenumber *m* = 2 and have typical amplitude of O(0.01) kg/m^3^. The gravitational anomalies are largely dominated by the two spherical harmonics, 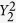 and 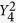 and have magnitude O(1) mgal.

**Figure 2:**
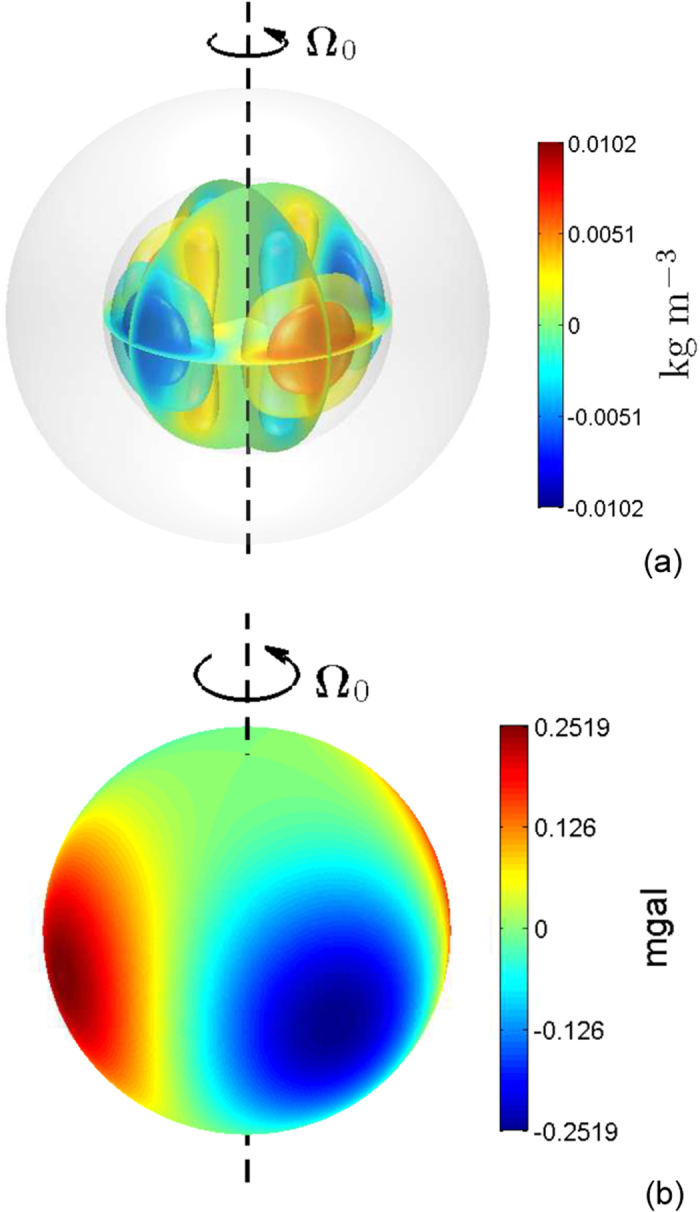
(**a**) Density anomalies in the metallic hydrogen region induced by convective motion with amplitude 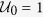 m/s and azimuthal wavenumber *m*_0_ = 2 in the rotationally distorted Jupiter and (**b**) the corresponding radial gravitational anomalies near the outer bounding surface of Jupiter. The metallic-molecular interface in this case is located at equatorial radius 0.6*R*_*e*_.

Results for a deeper dynamo model with depth parameter *H* = 0.4*R*_*e*_ are given in Fig. 2. Although the convection-induced density anomalies are still characterized by azimuthal wavenumber *m* = 2 with typical amplitude O(0.01) kg/m^3^, the corresponding radial gravitational anomalies near the outer bounding surface of Jupiter are only O(0.1) mgal. This reduction is caused by the deep location of the convective motion and the small volume of the metallic dynamo region.

**Figure 3:**
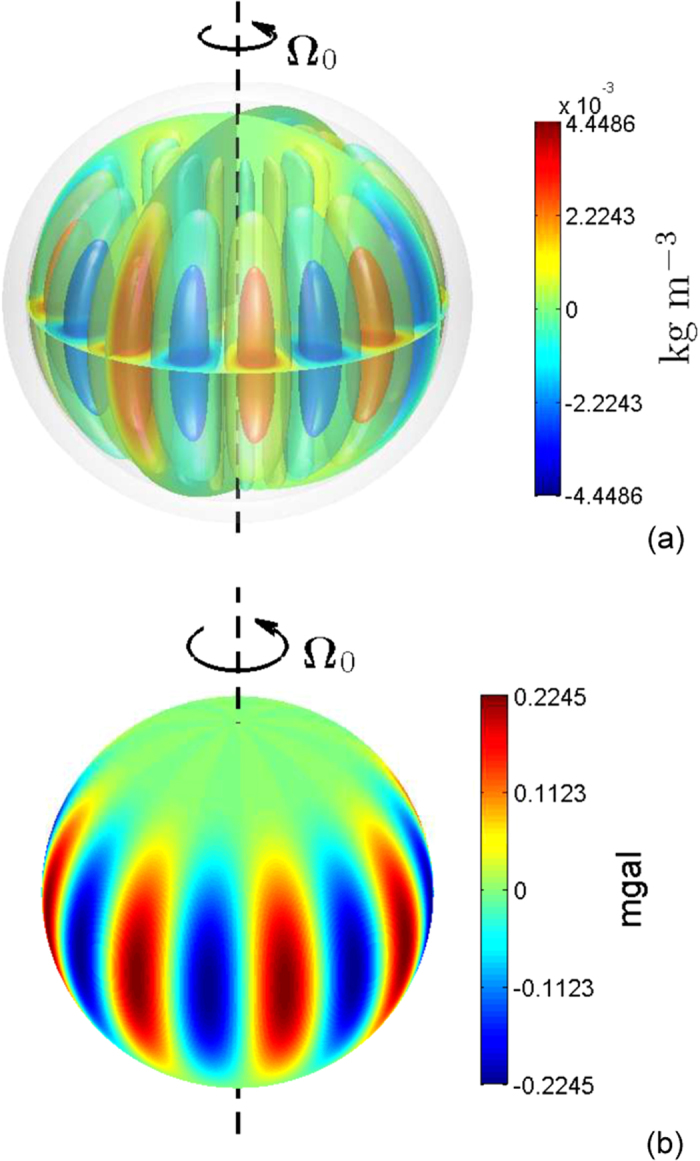
(**a**) Density anomalies in the metallic dynamo region induced by convective motion with amplitude 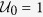 m/s and azimuthal wavenumber *m*_0_ = 8 in the rotationally distorted Jupiter and (**b**) the corresponding radial gravitational acceleration near the outer surface of Jupiter. The metallic-molecular interface is at the equatorial radius 0.9*R*_*e*_.

The convective motions of a weak-field Jovian dynamo in the metallic hydrogen-helium region would have a small horizontal scale^8^. In this case, we expect that the induced external gravitational signature would be weaker even with the same amplitude 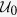 because the signature represents a globally average quantity. Accordingly, we carry out a computation for the same amplitude 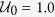 m/s but with a smaller horizontal scale of flow with azimuthal wavenumber *m*_0_ = 8. A three-dimensional view of the density anomalies in the metallic dynamo region is shown in Fig. 3(a) and the radial gravitational anomalies near the outer bounding surface are depicted in Fig. 3(b) for the depth parameter *H* = 0.1*R*_*e*_. The density and gravity anomalies are also characterized by *m* = 8 and have amplitudes O(0.001) kg/m^3^ and O(0.1) mgal. The gravitational anomalies have a structure dominated by 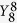 and 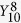. A small-scale convective flow produces a weak gravitational signature that would be more difficult to detect.

## Discussion

The results reported in this paper have significant implications for interpreting any nonaxisymmetric gravitational field detected by Juno or some future Jupiter orbiter and for understanding the physics of the convection-driven Jovian dynamo. Our fully three-dimensional model of gravitational sounding provides a possible way of probing the Jovian convective dynamo as an inverse problem, by comparing its effect on the external gravitational field to non-axisymmetric gravitational measurements. A dominant scale of the radial gravitational anomalies informs us about the corresponding scale of the deep convective flow and the amplitude of the radial gravitational anomalies reveals the amplitude of the flow and the depth where the Jovian dynamo is operating.

It is anticipated that the ongoing Juno spacecraft will achieve high precision with the expected noise level of O(10^−3^) mgal and relative uncertainty 10^−9^ for acceleration measurements^12^,^13^. Our gravitational sounding model predicts that nonaxisymmetric convective motion with typical amplitude 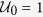 m/s produces radial gravitational anomalies with the magnitude O(1) mgal near the outer bounding surface of Jupiter and that non-axisymmetric motion with typical amplitude 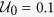 m/s produces anomalies with magnitude O(0.1) mgal. An accurate model of three-dimensional gravitational sounding together with detection of a nonaxisymmetric gravitational field will enable an important constraint on the amplitude, structure and location of the convection-driven Jovian dynamo. It is shown that high-precision measurements of the Jovian gravitational field can provide a complementary and effective way of probing the Jovian convective dynamo via three-dimensional gravitational sounding. Although our paper does not deal specifically with Saturn, our theory can also be applied to the gravitational measurements that will be made by the Cassini spacecraft at its end of mission.

## Methods

In our model of gravitational sounding, we regard some parameters of Jupiter as being well determined by observations while other parameters are treated as unknown. The equatorial and polar radii of Jupiter *R*_*e*_ and *R*_*p*_, –which are *R*_*e*_ = 71492 km and *R*_*p*_ = 66854 km –and the angular velocity Ω_*J*_ = 1.7585 × 10^−4^ s^−1^ and the total mass M_*J*_ = 1.8986 × 10^27^ kg will be regarded as known parameters. According to the models of Jupiter^7^,^14^, its interior, if the small rocky core is neglected, consists of two major parts: the outer molecular insulating envelope where the observed cloud-level zonal winds may be able to penetrate and the metallic hydrogen-helium region where the Jovian magnetic field is generated by its convective dynamo. It is well known that both the outer surface of the molecular envelope and the molecular-metallic interface are, because of rapid rotation and non-uniform density, non-spheroidal. A recent study^15^ shows, however, that the assumption of Roberts^16^ –that all equidensity surfaces for the hydrostatic equilibrium of a rapidly rotating Jupiter-like gaseous body are in the shape of oblate spheroids whose eccentricities are a function of the equatorial radius and whose axes of symmetry are parallel to the rotation axis–represents a reasonably accurate approximation. Our model assumes that both the molecular-metallic interface and the outer surface of the molecular envelope are in the shape of oblate spheroids with different eccentricities.

Our model also assumes that the Rossby number for the deep convective flow 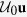 with the typical amplitude 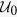 is small, viscous forces are much smaller than the Coriolis forces, the planet is rotating rapidly about the symmetry *z*-axis with the angular velocity 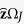 and is in a statistically steady state. The assumption leads to the following governing equations in the rotating frame of reference:


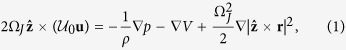



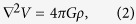



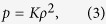



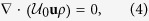


where 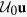 represents the velocity of convection, **r** denotes the position vector with origin at the center of figure, *p*(**r**) is the pressure and *ρ*(**r**) is the density, *K* is a constant, and *V* is the gravitational potential with *G* = 6.67384 × 10^−11^ m^3^ kg^−1^s^−2^ the universal gravitational constant. Although the effect of Lorentz forces produced by the magnetic field **B** cannot be explicitly included in the present model, it is implicitly reflected in the structure of the convective flow. According to the well-known magnetohydrodynamic theory for rapidly rotating systems^8^,^17^, a large horizontal scale of the flow marked by a small azimuthal wavenumber is likely associated with a Jovian dynamo with a strong magnetic field **B** while a small horizontal scale of the flow marked by a large azimuthal wavenumber is likely related to a Jovian dynamo with a weak magnetic field **B**.

Eqs (1, 2, 3, 4) with a parameterized convective flow 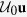 are to be solved subject to the two boundary conditions


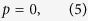



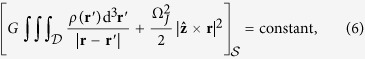


where 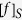 denotes the evaluation of *f* at the outer bounding surface 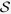 that is a priori unknown and 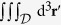 represents the volume integration over the domain enclosed by 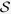.

For a mathematical description of the problem in the non-spherical metallic region, it is convenient to adopt cylindrical polar coordinates (*s, ϕ, z*) with *s* = 0 at the rotation axis of Jupiter, *z* = 0 at its equatorial plane and the corresponding unit vectors (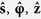). Eqs (1, 2, 3, 4) are then solved by a perturbation method making use of the expansions


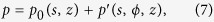



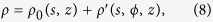



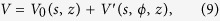


where the leading-order solution, [*p*_0_(*s, z*), *ρ*_0_(*s, z*), *V*_0_(*s, z*)], is two-dimensional and represents a hydrostatic state that accounts for the effect of rotational distortion but unaffected by the convective flow, and the next-order solution, *p*′(*s, ϕ, z*), *ρ*′(*s, ϕ, z*) and *V*′(*s, ϕ, z*), is fully three-dimensional and denotes the perturbations arising from the effect of the deep convection 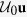. The expansions yield two problems that are mathematically coupled and inseparable. The leading-order problem determines the shape 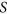 of Jupiter as well as the internal distribution *p*_0_(*s, z*), *ρ*_0_(*s, z*) and *V*_0_(*s, z*) in the hydrostatic equilibrium while the next-order problem, which is based on the leading-order solution, determines the perturbations *p*′(*s, ϕ, z*), *ρ*′(*s, ϕ, z*) and *V*′(*s, ϕ, z*) caused by the convective flow.

Substitution of the expansions into Eqs (1, 2, 3, 4) yields the leading-order problem governed by


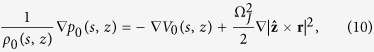



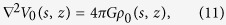



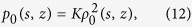


subject to the boundary conditions


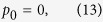



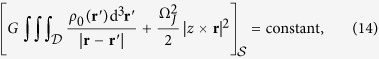


at the outer bounding surface 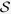 of Jupiter. Our calculations indicate that the difference between the *p*_0_ = 0 and *p*_0_ = 1 bar solutions is negligibly small. Eqs (10, 11, 12) together with the two boundary conditions can be solved to determine the outer bounding surface 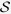 of the planet, the density distribution *ρ*_0_(*s, z*) and the corresponding gravitational potential *V*_0_(*s, z*). The leading-order problem is not the primary concern of this study.

The next-order problem, which gives rise to the density anomalies *ρ*′(*s, ϕ, z*) induced by the convective flow 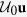 in the metallic dynamo region and the concomitant gravitational potential *V*′(*s, ϕ, z*), is governed by the equations


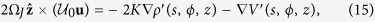



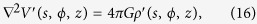



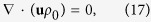


subject to the boundary condition


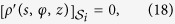


where 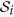 denotes a non-spherical interface between the molecular outer layer and the metallic dynamo region with the equatorial radius (*R*_*e*_ − *H*). For our model of gravitational sounding, we take a parameterized convective flow within the metallic region enclosed by 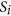 in the form


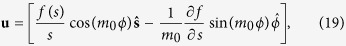


where *m*_0_ > 1 and 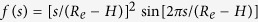. Our model of the parameterized flow is compatible with the Jupiter magnetic pole that is offset from its rotation axis by about 10 degrees. The parameterized convective flow approximately satisfies Eq. (17) and mimics the dynamically possible structure of convection under the rotational and magnetic influence^4^,^5^,^8^,^17^.

In our gravitational sounding of the Jovian interior, we compute, using a finite element method, the fully three-dimensional, convection-induced gravitational anomalies *g*′(*s, ϕ, z*) associated with the gravitational potential *V*′(*s, ϕ, z*). An accurate solution of gravitational sounding, together with the unprecedentedly high-precision gravitational measurements to be carried out by the Juno spacecraft, would determine the three key parameters that characterize the Jovian convective dynamo: the depth *H* below which the Jovian dynamo operates; the typical horizonal length 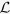 of the convective motion which maintains the Jovian dynamo; and the typical amplitude 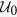 that must be azimuthally non-axisymmetric and sufficiently large to sustain the Jovian dynamo action.

## Additional Information

**How to cite this article**: Kong, D. *et al*. Using Jupiter’s gravitational field to probe the Jovian convective dynamo. *Sci. Rep.* **6**, 23497; doi: 10.1038/srep23497 (2016).
